# Dietary Intake of Nutrients and Lifestyle Affect the Risk of Mild Cognitive Impairment in the Chinese Elderly Population: A Cross-Sectional Study

**DOI:** 10.3389/fnbeh.2016.00229

**Published:** 2016-11-29

**Authors:** Yanhui Lu, Yu An, Jin Guo, Xiaona Zhang, Hui Wang, Hongguo Rong, Rong Xiao

**Affiliations:** ^1^School of Public Health, Capital Medical UniversityBeijing, China; ^2^Linyi Mental Health CenterLinyi, China

**Keywords:** dietary nutrients, vitamins, minerals, lipids, lifestyle, mild cognitive impairment, aging

## Abstract

Mild cognitive impairment (MCI) is a pre-clinical stage of Alzheimer’s disease afflicting a large number of the elderly throughout the world. However, modifiable risk factors for the onset and progression of MCI remain unclear. A cross-sectional study was performed to explore whether and how daily dietary nutrients intake and lifestyle impacted the risk of MCI in the Chinese elderly. We examined 2,892 elderly subjects, including 768 MCI patients and 2,124 subjects with normal cognition in three different Provinces of China. Dietary intake of nutrients were collected by using a 33-item food frequency questionnaire and calculated based on the Chinese Food Composition database. The MCI patients were first screened by Montreal Cognitive Assessment and then diagnosed by medical neurologists. Multivariate logistic regression and exploratory factor analyses were applied to identify and rank the risk factors. Three dietary nutrient intake combination patterns were identified as the major protective factors of MCI, with eigenvalues of 14.11, 2.26, and 1.51 and adjusted odds ratios (OR) of 0.77, 0.81, and 0.83 (*P* < 0.05), respectively. The most protective combination was featured with eight vitamins and six minerals, and OR for the third and fourth quartiles of these nutrients intake ranged from 0.48 to 0.74 (*P* < 0.05). Carotenoids, vitamin C, and vitamin B_6_ exhibited the highest protective factor loadings of 0.97, 0.95, and 0.92 (*P* < 0.05), respectively. Education, computer use, reading, and drinking represented the most protective lifestyle factors (OR = 0.25 to 0.85, *P* < 0.05), whereas smoking and peripheral vascular diseases were associated with higher (OR = 1.40 and 1.76, *P* < 0.05) risk of MCI. Adequate dietary intake of monounsaturated fatty acids and cholesterol were significantly associated with decreased risk of MCI. In conclusion, adequate or enhanced intake of micronutrients seemed to lower the risk of MCI in the Chinese elderly. In addition, improving education and lifestyle such as reading, computer use and moderate drinking might also help to decrease the risk of MCI.

## Introduction

Alzheimer’s disease (AD) has become a serious public health problem, resulting in tremendous economic loss and social burden. There will be an estimated 66 million people with AD or other dementias worldwide by the year 2030, and the number may reach 115 million by 2050 with an aging world population (Wortmann, 2012). Because mild cognitive impairment (MCI) is a precursory stage of AD (Janoutova et al., 2015), the MCI patients constitute a high-risk group that develop AD at a rate of 10–15% per year compared with the general population at a rate of 1–2% per year (Geda et al., 2013). Therefore, there has been a growing interest in preventive strategies to decrease the risk of MCI.

Epidemiological evidence has suggested lifestyle modification as a possible means of protecting against cognitive decline (Hugo and Ganguli, 2014). Likewise, dietary patterns may be of great importance in the prevention of cognitive decline in later life (Smith and Blumenthal, 2016). Several studies have indicated the actual benefits of early control of diets and lifestyle in lowering the rate of MCI (Eshkoor et al., 2015; Roberts et al., 2015; Lara et al., 2016). Meanwhile, prospective studies have shown that individuals with a “Mediterranean-like diets” were less likely to suffer from dementia or cognitive decline (Cao et al., 2015; Gardener et al., 2015). Similarly, two earlier studies by our laboratory have demonstrated that diet rich in marine products, fruit, vegetables, and vegetable juice could prevent cognitive decline in the elderly (Zhao et al., 2015; Dong et al., 2016). However, those studies examined only the association of daily intake of different foods, without considering the influence of individual nutrients on the risk of MCI. Furthermore, conclusions derived from those studies were inconsistent in illustrating the association between lifestyle (such as smoking, drinking or living status) and the risk of MCI (Durazzo et al., 2014; Roerecke and Rehm, 2014). While many factors might have contributed to such inconsistency, one of the limitations in those studies was the relatively small sample size from a single geographic area (Beijing). Another was that the collected data could have been analyzed using more appropriate programs such as exploratory factor analyses (Zhao et al., 2015; Dong et al., 2016). Notably, an important change in lifestyle of the elderly in China, like in other parts of the world, that could influence their cognitive function is the wide availability to personal computers. Therefore, we conducted the present cross-sectional analysis with 2,892 elderly subjects from three different areas in China and explored: (1) the association and importance of specific dietary nutrients intake with the risk of MCI; (2) the association of lifestyle including computer use with the risk of MCI; (3) the combined effects and relative importance of dietary nutrients and lifestyle on the risk of MCI.

## Materials and Methods

### Subjects

The subjects were recruited from February 2014 to March 2016 and selected from Chaoyang District, Beijing; Linyi, Shandong Province; and Jincheng, Shanxi Province. All the subjects were selected for the following inclusion criteria: 50–70 years old, capable of self-managing daily life, without self-recognized cognition dysfunction, and willing to participate in the study. Exclusion criteria for the subjects were as follows: individuals with serious illnesses (e.g., cancer, severe psychiatric disorders such as depression and schizophrenia, recent history of heart or respiratory failure, chronic liver or renal failure); individuals with condition known to affect cognitive function (e.g., a recent history of alcohol abuse, cerebral apoplexy and infarction); individuals with AD, Parkinson’s disease (PD) or long-term frequent intake of antidepressants and other medications for neurological diseases. The study protocol was approved by the Ethics Committee of Capital Medical University, Beijing (No.2013SY35). All participants were fully informed of the study, signed a written consent and allowed to terminate their participation at any time.

### Data Collection

Data on demographic and clinical characteristics, lifestyle and current use of medications were collected through face-to-face interviews by professionally trained nurses and researchers. The demographic characteristics included age, gender, body weight, height, household income, and education background. Body mass index (BMI) was calculated as weight (kg)/height^2^ (m^2^). The lifestyle covered smoking, alcohol consumption, working, reading and computer use. Smoking status were coded as non-smokers and smokers. Alcohol consumption was also dichotomized as current and non-drinkers. Work time was categorized into four groups (<40, 41–50, 51–60, and >60 h/week). History of chronic non-communicable diseases, including hypertension, diabetes and hyperlipidemia, was obtained using a self-guided questionnaire and ascertained on the basis of clinical diagnosis by physicians. Current medications use was recorded by self-reporting at the interview. Educational level was divided into low (illiterate, elementary school), middle (junior high school, senior high school, technical secondary school), and high (college and graduate school) according to average years of formal schooling (Sharp and Gatz, 2011).

### Dietary Assessment

A validated semi-quantitative food frequency questionnaire (FFQ) was used to estimate the consumption of a total of 33 items (whole grain, red meat, pork, beef, mutton, chicken, fruit and vegetables, legume and legume product, milk, fish, eggs, nuts, cooking oil, tea, etc.) (Block et al., 1992). All participants filled out the FFQ under the help of trained dietary interviewers and the information including the quantity of foods consumed as well as the consumption frequency (daily, weekly, monthly, yearly, or never). The quantity of food consumed was estimated using food models such as special charts and measuring rulers or cups to help in quantifying the consumed food. The dietary nutrients intake were calculated based on the quantity of total food intake and the China Food Composition Database (Yang et al., 2009).

### Assessment of Cognitive Function

Cognitive function was evaluated by Montreal Cognitive Assessment (MoCA) according to standard protocols. The total score of MoCA is 30 and the cut-off score commonly used for screening MCI in developed countries is 25/26 (Bischkopf et al., 2002). However, an increasing number of studies has demonstrated that the MoCA is strongly influenced by educational background and varying cut-offs stratified by educational level are recommended for the purpose of improving the effectiveness of the screening. Consequently, the cutoff score for MCI applied to the present study was 14 for illiterate individuals, 19 for individuals with 1–6 years of education, and 24 for individuals with 7 or more years of education. The same cutoff score proved to be appropriate for screening MCI in the Chinese elderly by a large population-based study (Lu et al., 2011). If the subjects met the above MCI criteria, they were arranged to visit the physicians for final diagnoses (Chertkow et al., 2008).

### Statistical Analyses

Data are presented as mean and standard deviation (SD), median [interquartile range (IQR)], or frequency (percentage). Characteristics were compared between MCI and normal cognition groups using factorial design ANOVA analyses for continuous variables and Cochran-Mantel-Haenszel χ^2^ test for categorical variables adjusted education levels. Multivariate logistic regression was used to estimate gender-specific associations between different nutrients intake or lifestyle and the risk of MCI. Exploratory factor analysis was used to derive food nutrients from the FFQ for all participants in the study. These factors were orthogonally rotated to generate uncorrelated factors. The retained number of factors was determined on the basis of several criteria including eigenvalue >1.0, hence clearly identifying major dietary nutrients. Factor scores were created for each subject as the linear combination of the dietary variables weighted by an equivalent of the factor loadings. All tests were performed using SAS software version 9.1.3 (SAS Institute, Inc., USA). Graphics were produced using the SAS and Excel (Microsoft, Inc., USA). All the statistical analyses were performed at the conventional two-tailed alpha level of 0.05.

## Results

### Subjects

A total of 768 MCI patients and 2,124 subjects with normal cognition were enrolled in the study. Sociodemographic characteristics and lifestyle of participants are presented in **Table 1**. Compared with normal group, MCI group had lower educational level (*P* = 0.0001), BMI (*P* = 0.0079) and frequencies of drinking (*P* = 0.0068), reading (*P* = 0.0001), and computer use (*P* = 0.0001). Besides, MCI group had longer working time (*P* = 0.0042) and higher morbidity of peripheral vascular diseases (PVDs) (*P* = 0.0019). The differences of dietary nutrients intake between the two groups are presented in **Table 2**. Except for polyunsaturated fatty acids (PUFAs) and vitamin E, the intake of other nutrients were significantly lower (*P* < 0.05) in MCI group than the control.

**Table 1:** Comparison of general characteristics between MCI patients and cognitively normal subjects.

Variables	Normal (*N* = 2124)	MCI (*N* = 768)	*P-*value
Gender; *n* (%)			0.1200
Male	1166 (54.9)	355 (46.2)	
Female	958 (45.1)	413 (53.8)	
Age, years	58.2 ± 5.3	58.3 ± 4.4	0.1900
BMI, kg/m^2^	25.6 ± 3.8	25.1 ± 3.2	0.0079
Educational level; *n* (%)			0.0001
Low	935 (44.1)	485 (63.1)	
Middle	598 (28.1)	211 (27.5)	
High	591 (27.8)	72 (9.4)	
Live situation; *n* (%)			0.6600
Not alone	2086 (98.2)	751 (97.8)	
Solitude	38 (1.8)	17 (2.2)	
Smoking; *n* (%)			0.0880
Yes	512 (24.1)	203 (26.4)	
No	1612 (75.9)	565 (73.6)	
Drinking; *n* (%)			0.0068
Yes	665 (31.3)	174 (22.7)	
No	1459 (68.7)	594 (77.3)	
Watching TV; *n* (%)			0.8900
Yes	2004 (94.4)	731 (95.2)	
No	120 (5.6)	37 (4.8)	
Reading; *n* (%)			0.0001
Yes	990 (46.6)	256 (33.3)	
No	1134 (53.4)	512 (66.7)	
Computer use; *n* (%)			0.0001
Yes	854 (40.2)	185 (24.1)	
No	1270 (57.8)	583 (75.9)	
Labor intensity; *n* (%)			0.0520
Mild	1903 (89.6)	656 (84.8)	
Moderate	169 (8.0)	81 (10.5)	
Strong	52 (2.4)	36 (4.7)	
Work time; *n* (%)			0.0042
1	1913 (90.1)	662 (86.2)	
2	109 (5.1)	61 (7.9)	
3	39 (1.8)	26 (3.4)	
4	63 (3.0)	19 (2.5)	
Hypertension; *n* (%)	630 (29.7)	242 (31.5)	0.2700
Diabetes; *n* (%)	297 (14.0)	124 (16.2)	0.0710
Hyperlipidemia; *n* (%)	392 (18.5)	147 (19.1)	0.2800
PVD; *n* (%)	78 (3.7)	46 (6.0)	0.0019
CHD; *n* (%)	126 (5.9)	57 (7.4)	0.2500


*BMI, body mass index; PVD, peripheral vascular diseases; CHD, coronary heart disease; TV, television.*

*Educational level: low (illiterate, elementary school), middle (junior high school, senior high school, technical secondary school), and high (college and graduate school).*

*Work time (h/week): 1, <40; 2, 41–50; 3, 51–60; and 4, >60.*

*Data are presented as mean ± SD or *n* (%). Characteristics were compared between the two groups using factorial design ANOVA analysis for continuous variables and Cochran-Mantel-Haenszel χ^2^ test for categorical variables adjusted education levels.*

**Table 2:** Comparison of dietary nutrients intake between MCI patients and cognitively normal subjects.

Variables	Normal (*N* = 2,124)	MCI (*N* = 768)	*P-*value
Cholesterol, mg/d	342.3 ± 193.5	278.5 ± 157.8	0.0001
MUFA, g/d	26.6 ± 13.9	22.2 ± 11.6	0.0001
PUFA, g/d	22.6 ± 12.0	22.6 ± 12.4	0.9900
Folic acid, μg/d	392.1 ± 115.5	351.4 ± 104.1	0.0001
Vitamin B_1,_ mg/d	1.48 ± 0.58	1.30 ± 0.48	0.0001
Vitamin B_2,_ mg/d	1.46 ± 0.70	1.21 ± 0.60	0.0001
Vitamin B_3,_ mg/d	21.1 ± 10.0	17.2 ± 8.2	0.0001
Vitamin B_6,_ mg/d	1.33 ± 0.80	1.02 ± 0.68	0.0001
Vitamin B_12,_ μg/d	2.02 ± 1.80	1.53 ± 1.48	0.0001
Vitamin C, mg/d	133.3 ± 74.5	109.4 ± 68.1	0.0001
Vitamin A, μg RAE/d	922.0 ± 522.6	723.1 ± 475.4	0.0001
Vitamin E, mg/d	37.2 ± 14.3	37.9 ± 15.6	0.2600
Iron, mg/d	38.4 ± 18.3	31.7 ± 15.6	0.0001
Magnesium, mg/d	452.9 ± 185.6	400.8 ± 163.9	0.0001
Zinc, mg/d	15.9 ± 6.6	13.5 ± 5.5	0.0001
Selenium, μg/d	53.1 ± 24.7	44.2 ± 21.1	0.0001
Copper, mg/d	3.75 ± 1.55	3.25 ± 1.36	0.0001
Manganese, mg/d	8.57 ± 3.29	7.68 ± 2.98	0.0001


*Data are presented as mean ± SD. Wilcoxon rank sum test was used to compare two groups.*

### Association between Lifestyle and Risk of MCI

In the adjusted multivariate logistic regression models of the overall sample analysis, drinking (OR = 0.85; 95% CI = 0.75, 0.97), reading (OR = 0.72; 95% CI = 0.60, 0.90), and computer use (OR = 0.57; 95% CI = 0.46, 0.70) were less likely to have MCI (**Table 3**). Furthermore, compared with low level of education, middle and high levels of education (OR = 0.69, 95% CI: 0.56, 0.85; OR = 0.25, 95% CI: 0.19, 0.34) were associated with less odds for MCI. Meanwhile, smoking and PVD were related with 1.40 (95% CI = 1.09, 1.80) and 1.76 (95% CI = 1.19, 2.59) times higher odds for MCI, respectively.

**Table 3:** Odds ratios (95% CI) for lifestyle related risk factors of MCI with different levels of adjustments.

	β^∗^	*P-*value^∗^	Odds ratio (95% CI)^∗^	Adjusted odds ratio (95% CI)^#^
Education				
Low			1.00	1.00
Middle	-0.3808	0.0001	0.68 (0.56–0.83)	0.69 (0.56–0.85)
High	-1.4279	0.0001	0.24 (0.18–0.32)	0.25 (0.19–0.34)
Live situation				
Not alone			1.00	1.00
Solitude	0.1210	0.4200	1.13 (0.84–1.51)	1.25 (0.85–1.85)
Smoking	0.4305	0.0001	1.54 (1.23–1.92)	1.40 (1.09–1.80)
Drinking	-0.1163	0.0410	0.89 (0.80–1.00)	0.85 (0.75–0.97)
Reading	-0.5120	0.0001	0.60 (0.50–0.71)	0.72 (0.60–0.90)
Computer use	-0.7140	0.0001	0.49 (0.41–0.59)	0.57 (0.46–0.70)
PVD	0.5370	0.0050	1.71 (1.18–2.49)	1.76 (1.19–2.59)
Labor intensity	0.3058	0.0001	1.42 (1.19–1.70)	1.20 (0.97–1.50)
Work time				
1			1.00	1.00
2	0.5538	0.0010	1.74 (1.25–2.42)	1.43 (0.98–2.09)
3	0.6728	0.0092	1.96 (1.18–3.25)	1.53 (0.86–2.72)
4	-0.1106	0.6781	0.90 (0.53–1.51)	0.69 (0.38–1.23)


*PVD, peripheral vascular diseases; CI, confidence interval; OR, odds ratio.*

*Worktime (h/week): 1, <40; 2, 41–50; 3, 51–60; and 4, >60.*

*^∗^Models adjusted for age and gender.*

***^#^**Model adjusted for age, gender, BMI, education, live situation, smoking, drinking, reading, computer use, labor intensity, working time, hypertension, diabetes, hyperlipidemia, PVD, and CHD.*

### Association between Intake of Dietary Nutrients and the Risk of MCI

Dietary intake of various nutrients were divided into four quartiles (Q1–Q4) as categorical variables (**Table 4**). When we applied logistic regression analysis to adjust for potential confounders and took the quartile of nutrient approximate to dietary reference intakes (DRIs, 2013 edition) as the reference, participants with Q3 and Q4 of cholesterol intake (OR = 0.63, 95% CI: 0.49, 0.82; OR = 0.54, 95% CI: 0.42, 0.70) and Q4 of MUFA intake (OR = 0.66, 95% CI: 0.50, 0.89) showed lower risks of MCI whereas Q2 of MUFA intake (OR = 1.33, 95% CI: 1.02, 1.74) had an increased risk. Other quartiles of nutrients intake significantly associated with MCI in the multivariate analysis were: Q1 of five vitamins (A, B_6_, B_12_, C, carotenoids) and Q4 of iodine with increased OR (1.29–1.91, *P* < 0.05); Q3 of vitamin B_3_ and three minerals (Mg, Zn, and Cu) with decreased OR (0.71–0.74, *P* < 0.05); Q4 of nine vitamins (A, B_1_, B_2_, B_3_, B_6_, B_12_, C, folic acid, and carotenoids) and six minerals (Zn, Mg, Fe, Se, Cu, and Mn) with lower OR (0.48–0.71, *P* < 0.05).

**Table 4:** Odds ratios (95% CI) for dietary nutrients intake related risk factors of MCI with different levels of adjustment.

	β^∗^	*P-*value^∗^	Odds ratio (95% CI)^∗^	Adjusted odds ratio (95% CI)^#^
Cholesterol				
Q1	0.096	0.363	1.10 (0.90–1.35)	1.07 (0.87–1.33)
Q2			1.00	1.00
Q3	-0.514	0.000	0.60 (0.46–0.77)	0.63 (0.49–0.82)
Q4	-0.826	0.000	0.44 (0.34–0.56)	0.54 (0.42–0.70)
MUFA				
Q1	0.281	0.028	1.33 (1.03–1.70)	1.16 (0.90–1.50)
Q2	0.458	0.001	1.58 (1.22–2.05)	1.33 (1.02–1.74)
Q3			1.00	1.00
Q4	-0.472	0.001	0.62 (0.47–0.83)	0.66 (0.50–0.89)
PUFA				
Q1	-0.111	0.387	0.90 (0.70–1.15)	0.79 (0.61–1.02)
Q2	0.186	0.160	1.21 (0.93–1.56)	1.06 (0.81–1.39)
Q3			1.00	1.00
Q4	0.044	0.743	1.05 (0.80–1.36)	0.95 (0.72–1.24)
Folic acid				
Q1	1.183	0.083	1.20 (0.98–1.48)	1.12 (0.64–1.97)
Q2			1.00	1.00
Q3	-0.207	0.101	0.81 (0.63–1.04)	0.78 (0.48–1.26)
Q4	-0.869	0.000	0.42 (0.33–0.54)	0.51 (0.33–0.79)
Vitamin B_6_				
Q1	0.286	0.006	1.33 (1.08–1.64)	1.91 (1.02–3.58)
Q2			1.00	1.00
Q3	-0.374	0.004	0.69 (0.53–0.89)	0.63 (0.39–1.05)
Q4	-0.901	0.000	0.41 (0.32–0.52)	0.54 (0.35–0.89)
Vitamin B_12_				
Q1	0.359	0.001	1.43 (1.16–1.76)	1.37 (1.11–1.70)
Q2			1.00	1.00
Q3	-0.086	0.502	0.92 (0.72–1.18)	0.98 (0.76–1.26)
Q4	-0.526	0.000	0.59 (0.47–0.75)	0.70 (0.54–0.90)
Vitamin A				
Q1	0.257	0.015	1.29 (1.05–1.59)	1.30 (1.05–1.61)
Q2			1.00	1.00
Q3	-0.194	0.126	0.82 (0.64–1.06)	0.94 (0.72–1.21)
Q4	-0.764	0.000	0.47 (0.37–0.60)	0.58 (0.45–0.74)
Carotenoids				
Q1	0.257	0.015	1.29 (1.05–1.59)	1.31 (1.06–1.62)
Q2			1.00	1.00
Q3	-0.297	0.022	0.74 (0.58–0.96)	0.81 (0.63–1.05)
Q4	-0.684	0.000	0.51 (0.40–0.64)	0.62 (0.48–0.79)
Vitamin E				
Q1			1.00	1.00
Q2	-0.165	0.140	0.85 (0.68–1.06)	0.88 (0.70–1.72)
Q3	-0.295	0.032	0.74 (0.59–0.98)	0.82 (0.61–1.09)
Q4	0.102	0.384	1.11 (0.88–1.39)	1.19 (0.93–1.52)
Vitamin C				
Q1	0.392	0.000	1.48 (1.20–1.82)	1.44 (1.17–1.78)
Q2			1.00	1.00
Q3	-0.116	0.362	0.89 (0.69–1.14)	0.97 (0.75–1.25)
Q4	-0.637	0.000	0.53 (0.42–0.67)	0.65 (0.50–0.84)
Vitamin B_1_				
Q1	0.118	0.266	1.13 (0.91–1.39)	1.09 (0.88–1.36)
Q2			1.00	1.00
Q3	-0.076	0.538	0.93 (0.73–1.18)	1.03 (0.80–1.32)
Q4	-0.836	0.000	0.43 (0.34–0.56)	0.54 (0.42–0.70)
Vitamin B_2_				
Q1	0.102	0.331	1.11 (0.90–1.36)	1.09 (0.88–1.35)
Q2			1.00	1.00
Q3	-0.330	0.009	0.72 (0.56–0.92)	0.80 (0.62–1.03)
Q4	-0.907	0.000	0.40 (0.32–0.52)	0.50 (0.38–0.64)
Vitamin B_3_				
Q1	0.154	0.142	1.17 (0.95–1.43)	1.16 (0.94–1.44)
Q2			1.00	1.00
Q3	-0.455	0.000	0.63 (0.49–0.82)	0.71 (0.55–0.92)
Q4	-0.954	0.000	0.39 (0.30–0.49)	0.48 (0.37–0.63)
Iron				
Q1	0.193	0.066	1.21 (0.99–1.49)	1.18 (0.96–1.46)
Q2			1.00	1.00
Q3	-0.293	0.021	0.75 (0.58–0.96)	0.80 (0.62–1.03)
Q4	-0.936	0.000	0.39 (0.31–0.50)	0.48 (0.37–0.63)
Magnesium				
Q1	0.134	0.203	1.14 (0.93–1.41)	1.12 (0.90–1.38)
Q2			1.00	1.00
Q3	-0.403	0.002	0.67 (0.52–0.86)	0.74 (0.57–0.97)
Q4	-0.709	0.000	0.49 (0.39–0.62)	0.60 (0.47–0.77)
Zinc				
Q1	0.107	0.306	1.11 (0.91–1.37)	1.12 (0.90–1.38)
Q2			1.00	1.00
Q3	-0.448	0.001	0.64 (0.50–0.82)	0.73 (0.56–0.95)
Q4	-0.931	0.000	0.39 (0.31–0.51)	0.50 (0.38–0.64)
Selenium				
Q1	0.336	0.009	1.40 (1.09–1.80)	1.24 (0.96–1.60)
Q2	0.517	0.000	1.68 (1.29–2.98)	1.48 (1.13–1.94)
Q3			1.00	1.00
Q4	-0.491	0.001	0.61 (0.46–0.82)	0.71 (0.53–0.96)
Copper				
Q1			1.00	1.00
Q2	-0.082	0.437	0.92 (0.75–1.13)	0.98 (0.79–1.23)
Q3	-0.548	0.001	0.58 (0.44–0.76)	0.71 (0.53–0.95)
Q4	-0.848	0.001	0.43 (0.33–0.55)	0.60 (0.46–0.80)
Manganese				
Q1			1.00	1.00
Q2	-0.072	0.500	0.93 (0.76–1.15)	1.00 (0.80–1.25)
Q3	-0.371	0.005	0.69 (0.53–0.90)	0.84 (0.64–1.12)
Q4	-0.804	0.001	0.45 (0.35–0.58)	0.64 (0.49–0.85)
Iodine				
Q1	0.389	0.000	1.48 (1.19–1.83)	0.82 (0.46–1.44)
Q2			1.00	1.00
Q3	-0.362	0.011	0.70 (0.53–0.92)	0.85 (0.64–1.13)
Q4	0.379	0.001	1.46 (1.18–1.81)	1.29 (1.03–1.62)


*The intake of all dietary nutrients were divided into four quartiles as categorical variables, with the quartiles closest to dietary reference intakes (DRIs) as the respective referent group.*

*^∗^Models adjusted for age and gender.*

***^#^**Model adjusted for age, gender, BMI, education, live situation, smoking, drinking, Reading, Computer use, labor intensity, work time, hypertension, diabetes, hyperlipidemia, PVD, and CHD.*

### Gender-Specific MCI Risk

**FIGURE 1:**
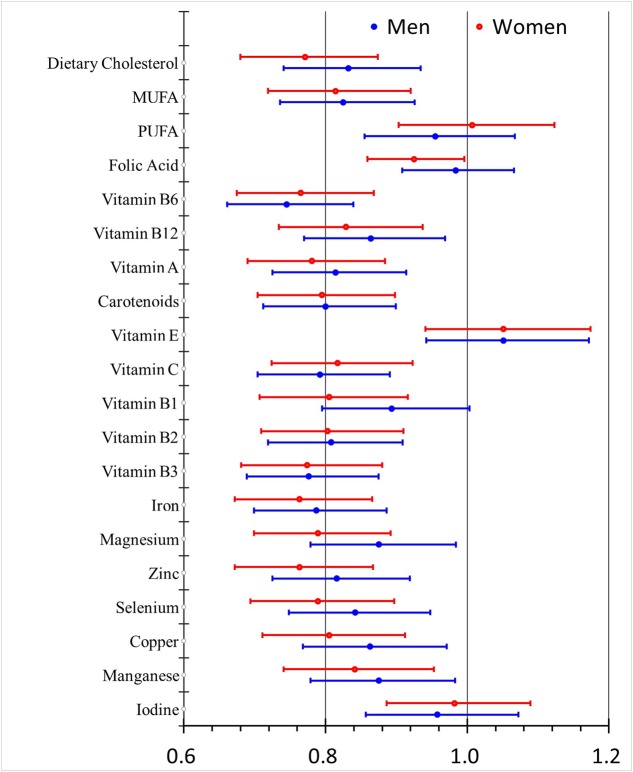
**Odds ratios (95% CI) for dietary nutrients intake-related risk factors of MCI with different adjustment levels in men and women**.

**FIGURE 2:**
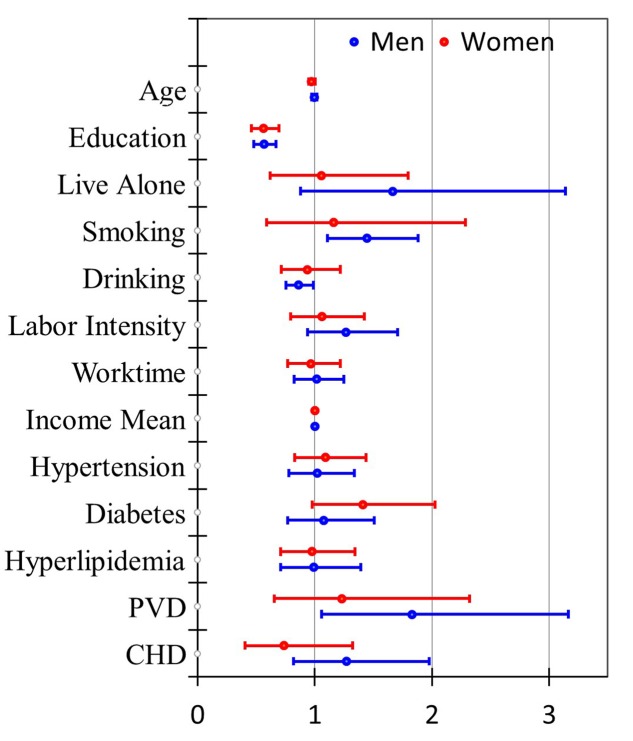
**Odds ratios (95% CI) for lifestyle-related risk factors of MCI with different adjustment levels in men and women**.

Compared with the pooled gender analysis, the males and females shared similar OR values for dietary intake of all assayed nutrients except for folic acid which was detected as a protective factor only in the females. Besides, vitamin B_1_ and magnesium seemed to be stronger protective factors in the females (**Figure 1**). The effects of education were consistent in both genders (**Figure 2**) while smoking, drinking and PVD were significant only in the males, with two as the risk factors and one as the protective factor.

### Ranking of Major Risk Factors

Three major factor patterns were identified by the exploratory factor analysis, with eigenvalues of 14.11, 2.26, and 1.51 and adjusted OR of 0.77, 0.81, and 0.83, respectively (**Table 5**). The first pattern was characterized as “vitamin and mineral pattern” that included eight vitamins and six minerals. The three highest factor loadings were carotenoids, vitamin C, and vitamin B_6_ as 0.97, 0.95, and 0.92, respectively. Vitamin B_1_ had the lowest loading factor of 0.47. The second pattern was labeled as “fatty acid pattern,” in which MUFA and dietary cholesterol had the highest loading factors of 0.86 and 0.53, respectively. The third pattern was marked as “mineral pattern” that was featured by manganese (Mn) and magnesium (Mg) with the two highest loading factors of 0.64 and 0.57, respectively, second to only vitamin B_1_ (0.65).

**Table 5:** Factor loadings for rotated factor patterns of daily intake of dietary nutrients.

Nutrients	Pattern 1	Pattern 2	Pattern 3
Carotenoids	0.97	…	…
Vitamin C	0.95	…	…
Vitamin B_6_	0.92	…	…
Folic acid	0.87	…	…
Vitamin A	0.84	…	…
Iron	0.82	…	0.44
Vitamin B_2_	0.81	…	0.33
Copper	0.71	…	0.54
Manganese	0.67	…	0.64
Magnesium	0.66	…	0.57
Zinc	0.64	0.42	0.52
Vitamin B_3_	0.62	0.47	0.47
Selenium	0.52	0.44	0.45
Vitamin B_1_	0.47	0.51	0.65
MUFA	…	0.86	…
Cholesterol	…	0.53	…
PUFA	…	…	…
Vitamin E	…	…	…
Vitamin B_12_	…	…	…
Iodine	…	…	…
Eigenvalue	14.11	2.26	1.51
Odds ratio^∗^	0.72 (0.67–0.77)	0.76 (0.71–0.82)	0.75 (0.70–0.81)
Adjusted OR^#^	0.77 (0.71–0.83)	0.81 (0.75–0.87)	0.83 (0.76–0.89)


*Factor pattern 1: vitamin and mineral pattern; Factor pattern 2: fatty acid pattern; Factor pattern 3: mineral pattern. Factor loadings less than 0.40 are not shown.*

*OR, odds ratio.*

*^∗^Models adjusted for age and gender.*

***^#^**Model adjusted for age, gender, BMI, education, live situation, smoking, drinking, Reading, Computer use, labor intensity, work time, hypertension, diabetes, hyperlipidemia, PVD, and CHD.*

## Discussion

One of the most interesting findings of present study is the identification of three major dietary factor patterns with eigenvalues ranging from 14.11 to 1.51 and adjusted OR of 0.77 to 0.83 toward the risk of MCI in the Chinese elderly from three different regions. A panel of eight vitamins and six mineral elements accounted for the vitamin and mineral pattern of factor 1 and showed the strongest association in protecting against the development of MCI. Among the 14 nutrients, five of them including carotenoids, vitamins A, C, and B_6_, and folic acids depicted the strongest association. Removing these five nutrients came up the factor pattern three that included six elements (Fe, Cu, Mn, Mg, Zn, and Se) and three B vitamins (B_1_, B_2_, B_3_). This group of nutrients was described as mineral pattern and showed the lowest eigenvalue and the least reduction in OR. Compared with the factor pattern 1, the factor pattern 2 included two unique lipid nutrients (MUFA and cholesterol) and shared only four other nutrients (Zn, Se, vitamins B_1_ and B_3_). The eigenvalue and OR values of the factor pattern 2 were very similar to those of the factor pattern 3. Overall, four nutrients (Zn, Se, and vitamins B_1_ and B_3_) appeared in all the three patterns, whereas the two lipid nutrients displayed only in the factor pattern 2. It is very intriguing that four nutrients (PUFA, vitamins E and B_12_, and iodine) were excluded from any of these three patterns.

Dietary nutrients serve as controllable or modifiable environmental factors possibly contributing to the development of MCI. It has been reported that dietary patterns and body nutritional status might affect cognitive function (Smith and Blumenthal, 2016). Previous studies have provided evidence that higher adherence to a Mediterranean diet was associated with slower cognitive decline or reduced risk of MCI (Feart et al., 2015). The identified ‘cognition-protective’ nutrients combination was manifested with higher intake of fresh fruit and vegetables, fish, and whole grains, and lower intake of sweets, high-fat dairies, and processed butter and meat (Berti et al., 2015). Our previous study also observed the protective effects of diets rich in fish and vegetables on the cognitive function in the elderly (Zhao et al., 2015; Dong et al., 2016). In fact, the revealing of factor pattern 1 in the present study, containing the complete panel of 14 micronutrients as the strongest protective matrix, was consistent with our and others’ previous observations regarding the cognition-protective nutrient combination and dietary pattern. These results indicated that diets or foods rich in vitamins and minerals, in particular the five top protective nutrients (carotenoids, vitamins C, B_6_, and A, and folic acid) could benefit the cognition preservation in the elderly. The presence of vitamins B_1_ and B_3_ in all the three protective nutrient combination patterns implied an important role of them in that regard. Recently, intake of total B vitamins have been shown to be associated with better cognitive function in the cognitively impaired elderly, especially in AD patients (Kim et al., 2014; Li et al., 2014). Partial mechanisms underlying this association have been proposed including antioxidant defense and lower occurrence of methylation reactions in central nervous system (Haan et al., 2007; Araujo et al., 2015).

In the current study, we have illustrated that dietary intake of certain nutrients were associated with dose (quartile)-dependent changes in OR values of MCI compared with the respective reference quartiles. The significantly elevated OR for Q1 of vitamin A, B_6_, B_12_, C, and carotenoids intake, compared with Q2, suggested that inadequate dietary intake of these nutrients that were lower than DRIs could increase the risk of developing MCI in the elderly. It is also worth noting that the estimated dietary intake of selenium (Se) in the study population in the present study was fairly low (44–53 μg/day). This led to two outcomes: (1) the reference quartile was actually Q3 so that 50% of the subjects did not consume adequate Se; (2) subjects with Q2 of the Se intake had increased risk of MCI while those with Q4 of the Se intake had decreased risk.

The relationship between poor nutritional status and cognitive impairments was previously described. Individuals with low Dietary ingestion or blood concentration of vitamin C and B_12_ performed poorly on the cognitive function test (O’Leary et al., 2012). Similar results were reported in a study of old subjects living in Manhattan (Luchsinger et al., 2007). These results were partly in accordance with our findings which imply significant associations of inadequate dietary intake of certain nutrients with increased risks of MCI in the Chinese elderly. Since oxidative stress has been implicated as one of the primary causes of cognitive impairments (Farr et al., 2014), antioxidant nutrients such as vitamin C are efficient in alleviating oxidative stress at the initial stage of cognitive impairments (Dror et al., 2014). Indeed, several studies have reported protective effects of vitamins C on the development of AD (Arlt et al., 2012; Mohajeri et al., 2015). This supports our finding that adequate vitamin C intake was one of the strongest protective micronutrients in the pattern analysis.

Interestingly, we have revealed the unique combination of two lipid nutrients (MUFA and cholesterol) with micronutrients (Zn and vitamins B_1_ and B_3_) as the protective nutrient matrix pattern 2. The effects of dietary intake of fatty acids and cholesterol on cognitive impairments have received particular attention, but the results are inconsistent (Grant, 2003; Del Parigi et al., 2006). Among 6,183 older participants in the Women’s Health Study by using serial cognitive tests found that a higher MUFA intake was related to a better global cognition and verbal memory (Okereke et al., 2012). A recent animal experiment has examined the underlying mechanism of the protective effects of cholesterol and indicated that a high-cholesterol diet enriched with polyphenols could improve cognitive function by down-regulating brain cholesterol levels and neurodegenerative-related protein expression (Kuo et al., 2015). Since our study was a cross-sectional analysis, the derived significant association between present consumptions of cholesterol or MUFA and MCI might not actually reflect the impact of past cholesterol and MUFA intake on the current risk or status of MCI. Therefore, the potential mechanism of these dietary lipids in preserving cognitive function in the elderly need to be further tested in randomized controlled study or longitudinal studies.

Our study has also analyzed a number of lifestyle-related factors to the risk of MCI. The illustrated protective factors consist of education, computer use, reading, and drinking, whereas the risk factors include smoking and PVD. Other studies have reported similar protective roles of education and reading against the development of MCI (Luck et al., 2010; Eshkoor et al., 2015; Zhao et al., 2015; Dong et al., 2016; Li et al., 2016). Silbert et al. (2016) have focused on the association between computer uses and volumetric markers of neurodegeneration on brain MRI. They have found that cognitively intact volunteers with less daily computer use showed decreased gray matter density in the temporal lobes and bilateral hippocampi, which may shed light on the potential mechanism underlying the association. Despite the protective role of drinking in decreasing risk of MCI shown in our present study, systematic reviews have summarized major discrepancies regarding the effects of alcohol consumption on AD, probably due to variations of quantity and/or frequency of drinking (Piazza-Gardner et al., 2013; Roerecke and Rehm, 2014). Controversy also exists on the association of smoking with the risk of MCI (Durazzo et al., 2014; Deochand et al., 2015). In the present study, smoking was a strong risk factor (OR = 1.40, 95% CI: 1.09, 1.80) of MCI. Potentially, smoking may cause detrimental effects on memory via oxidative stress induced by decreasing plasma glutathione and serum superoxide dismutase activity and vitamin C concentration (Kim et al., 2003, 2004). The resultant oxidative injuries promote Aβ deposition and abnormal tau phosphorylation (Giunta et al., 2012), thus contributing to the development of AD. Notably, drinking and smoking associated with the risk of MCI remained significant only in the male subjects in our study. This was probably because fewer females were actually drinking or smoking. Besides, PVD was another strong risk factor only in the male subjects. Since PVD often coexists with aging, smoking, and obesity (Cassar et al., 2010), the gender-specific differences of PVD in MCI warrants further research.

### Strengths and Limitations

Strengths of the present study are the relatively large sample size consisted of 2,892 participants recruited from three different regions of China, which was adequate for longitudinal studies. The health examination records, stored in the Physical Examination Centers, could provide an objective, accurate, and relatively large dataset for potential confounders. Furthermore, the statistical analyses adopted in this study enabled us to reveal three unique dietary nutrient intake combination patterns as the major protective factors in lowering the risk of MCI in the elderly.

However, the self-reporting derived FFQ data collected from the present study might suffer from recall bias, although the FFQ was one of the most commonly used methods to assess dietary patterns and to calculate the quantity of nutrient consumption. Despite a relatively large sample size, our study was still a cross-sectional analysis. Therefore, longitudinal studies with several follow-up or randomized controlled trials will be needed to explore and better define the cause-effect relationships between dietary nutrients intake or lifestyle and MCI. Equally important, mechanistic studies should be conducted in animals or *in vitro* to elucidate the molecular pathway and physiological regulation underlying the observed associations.

## Conclusion

Our study revealed three dietary nutrients intake patterns as the major protective factors against the development of MCI in the Chinese elderly. Adequate or enhanced daily intake of carotenoids, vitamin C, and vitamin B_6_ ranked as the most protective factors in preserving the cognition in the old Chinese subjects. Improving lifestyle with more education, computer use, reading, and moderate drinking were also protective against, whereas smoking and PVD were potentiating the risk of MCI. Future research is required to confirm and explain the positive effects of dietary MUFA and cholesterol intake on lowering the risk of MCI.

## Ethics Statement

The study protocol was in accordance with the Declaration of Helsinki and ethically approved by the Ethics Committee of Capital Medical University (2013SY35). The consent contains project title, investigators and other project personnel, introduction to project and invitation to participates, what this project is about and why it is being undertaken, project and researcher interests, what participation will involve, participant rights and interests, who to contact. Outline realistically any potential risks and relative preventive arrangements are in place.

## Author Contributions

RX conceived and designed the study, YL collected the data, performed the analyses and wrote the manuscript. YA, JG, XZ, HW, and HR helped collect and analyze the data. All authors read and approved the final manuscript.

## Conflict of Interest Statement

The authors declare that the research was conducted in the absence of any commercial or financial relationships that could be construed as a potential conflict of interest.
